# *Argonaute2* Is Essential for Mammalian Gastrulation and Proper Mesoderm Formation

**DOI:** 10.1371/journal.pgen.0030227

**Published:** 2007-12-28

**Authors:** Reid S Alisch, Peng Jin, Michael Epstein, Tamara Caspary, Stephen T Warren

**Affiliations:** 1 Department of Human Genetics, Emory University School of Medicine, Atlanta, Georgia, United States of America; 2 Department of Biochemistry, Emory University School of Medicine, Atlanta, Georgia, United States of America; 3 Department of Pediatrics, Emory University School of Medicine, Atlanta, Georgia, United States of America; University of California San Francisco Diabetes Center, United States of America

## Abstract

Mammalian *Argonaute* proteins (EIF2C1−4) play an essential role in RNA-induced silencing. Here, we show that the loss of *eIF2C2* (*Argonaute2* or *Ago2*) results in gastrulation arrest, ectopic expression of *Brachyury (T),* and mesoderm expansion. We identify a genetic interaction between *Ago2* and *T,* as *Ago2* haploinsufficiency partially rescues the classic *T/+* short-tail phenotype. Finally, we demonstrate that the ectopic *T* expression and concomitant mesoderm expansion result from disrupted fibroblast growth factor signaling, likely due to aberrant expression of *Eomesodermin*. Together, these data indicate that a factor best known as a key component of the RNA-induced silencing complex is required for proper fibroblast growth factor signaling during gastrulation, suggesting a possible micro-RNA function in the formation of a mammalian germ layer.

## Author Summary

Gastrulation is a developmental phase that delineates the three embryonic germ layers: ectoderm, endoderm, and mesoderm. The gene *Brachyury* is essential for mesoderm development, and short-tail mice, which were later found to be carrying a *Brachyury* mutation, have been known since 1927. In this study, we found a genetic interaction between *Brachyury* and another gene in mouse, *Argonaute2*. We show that the loss of *Argonauate2,* a necessary component of a recently appreciated pathway of gene regulation called RNA interference, results in embryonic death during gastrulation, abnormal expression of *Brachyury,* and expansion of the mesoderm layer. This suggests that *Argonaute2* is important in early development and in regulating *Brachyury* function. Consistent with this conclusion, we found that mice simultaneously carrying mutations in both *Argonaute2* and *Brachyury* have significantly longer tails than mice with only a *Brachyury* mutation. A closer look at other genes involved in mesoderm development revealed that a disruption in fibroblast growth factor signaling may explain the mesoderm expansion in mice carrying the *Argonaute2* mutation. Together this work demonstrates that a factor best known as a key component of RNA interference is required for the formation of a mammalian germ layer.

## Introduction

*Argonaute* proteins comprise a highly conserved gene family necessary for a range of physiological and developmental processes. These proteins are defined by the presence of PAZ and PIWI domains, which modulate protein−protein interactions, nucleic acid binding, and, in some cases, mRNA cleavage [1–5]. *Argonaute* proteins serve as scaffolds for target-mRNA recognition by short regulatory guide RNAs during the process of RNA interference (RNAi) [6]. The *Argonaute* family was initially linked to RNAi-related phenomena through genetic studies in Caenorhabditis elegans [7] and has since been shown to play a gene-silencing role in plants, yeast, and flies [8–10]. Members of the mammalian *Argonaute* family associate with micro-RNAs in the RNA-induced silencing complex (RISC), indicating a post-transcriptional gene regulation role in mammals [6]. In the mouse, loss of a single *Argonaute* family member, *eIF2C2* (*Argonaute2* or *Ago2*), disrupts RISC activity and gives rise to several midgestational developmental abnormalities, including failed neural tube closure, mispatterning of anterior structures, and cardiac malformations [11]. These studies demonstrated that AGO2 has a unique function distinct from its paralogs in the RISC, which indicates the absence of full paralog redundance. However, the specific role played by AGO2 during mammalian development remains unclear. To characterize this role, we investigated *Ago2*-null embryos during gastrulation and found that *Ago2* is required for proper fibroblast growth factor (FGF) signaling and mesoderm formation. We further determine that *Ago2* haploinsufficiency partially rescues the classic *T/+* short-tail phenotype [12], which is consistent with *Ago2* residing in a previously mapped interval shown to modify *T* [13]. Together, these data reveal a genetic interaction between *Ago2* and *T* and indicate that AGO2 is essential to the formation of a mammalian germ layer.

## Results/Discussion

**Figure 1:**
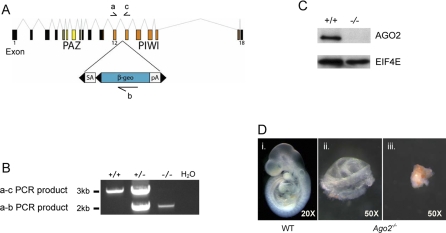
Characterization of the *Ago2* Disruption in ES Cell Clone RRE192 (A) Insertion of the gene-trap vector into intron 12 of the mouse *Ago2* locus. Exons 1, 12, and 18 are labeled. Exons encoding the PAZ domain are shown in yellow, and exons encoding the PIWI domain are shown in orange. The insertion cassette contains a splice acceptor (SA), a fusion of the β-galactosidase and neomycin phosphotransferase coding sequences (β-geo), and a polyadenylation signal (pA). FRT and loxP sites are denoted as black triangles. The relative location of primers used for genotyping are shown as half-arrows and are labeled a, b, and c. (B) The genotypes of embryos from heterozygous intercrosses. Shown is a gel displaying PCR products using primers a and c, identifying the normal allele, and primers a and b, identifying the interrupted allele. The PCR loaded into the water lane lacked template DNA and acts as a negative control. +/+ represents wild-type; +/– represents *Ago2* heterozygote; –/– represents *Ago2* homozygous mutant. (C) Western blot analysis of AGO2 in e7.5 wild-type (+/+) and *Ago2* homozygous mutant (–/–) embryos. EIF4E was used as a loading control. (D) Variable phenotype of *Ago2* homozygous *(Ago2^–/–^)* mutant embryos at e9.5. Shown here are three littermates. The variable *Ago2^–/–^* phenotypes included deciduas containing only embryonic remnants (ii and iii). This phenotype also was variable as either remnants of embryoid structures (ii) or as cell masses lacking obvious embryonic development (iii). Note the size magnification of the embryonic remnants, as they are much smaller than the wild-type (WT) littermate (i).

We explored the role of AGO2 in early mammalian development using gene-trapped embryonic stem cells to generate a mouse line that transmits an interrupted *Ago2* allele without an obvious heterozygous phenotype. The interrupted *Ago2* allele was characterized, and primers were designed to distinguish wild-type from mutants by genotype (Figure 1A and 1B). This disruption deletes most of the PIWI domain and results in an apparent functional null allele [11] (Figure 1A and 1C). Full-term litters from heterozygous intercrosses did not yield homozygous *(Ago2^–/–^)* offspring. At embryonic day 9.5 (e9.5), we observed two classes of null embryo: intact embryos with assorted morphological phenotypes, such as the neural tube and cardiac malformations that are consistent with the earlier findings of Liu and colleagues ([11]; unpublished data), and embryonic remnants (Figure 1D). Unexpectedly, however, intact e9.5 null embryos were observed in numbers significantly lower than predicted based on genetic ratios (12/134; *p* < 0.0001; Table 1). Because intact null embryos were recovered in the appropriate genetic ratios during gastrulation (i.e., at e7.5; Table 1), *Ago2* plays an important role at an earlier stage of development than previously reported [11].

**Table 1:**
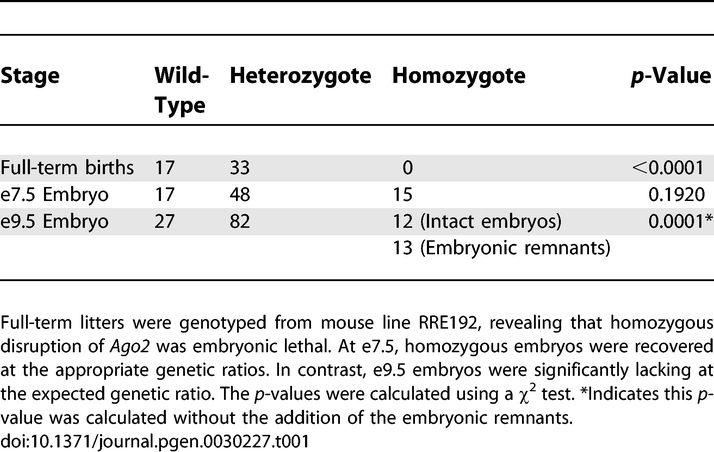
Full-term and Embryonic Litter Numbers From *Ago2* Heterozygous Crosses

**Figure 2:**
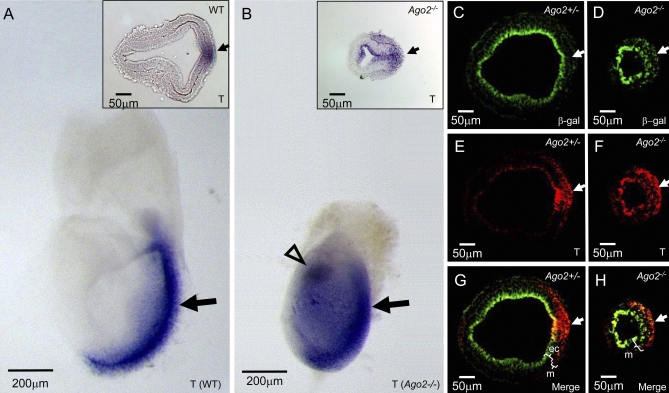
The Homozygous Disruption of *Ago2* Results in an Expansion of *T* Expression and Mesoderm Formation (A, B) Whole-mount in situ hybridization using an antisense probe against *T* on e7.5 wild-type (A) and *Ago2* ^–/–^ (B) embryo littermates. The *Ago2* ^–/–^ embryos exhibit an expansion of the primitive streak (block-arrow). Note that *Ago2* ^–/–^ e7.5 embryos are smaller and rounder than wild-type, suggesting aberrant growth. The scale bar represents 200 μm. (A, B, insets) Sections from whole-mount in situ hybridized e7.5 embryos. Shown are representative wild-type (A, inset) and *Ago2* ^–/–^ embryos (B, inset). The scale bar represents 50 μm. (C−H) Paraffin sections from *Ago2^+/^* ^–^ (C, E, G) and *Ago2* ^–/–^ (D, F, H) e7.5 embryos were stained with antibodies against β-galactosidase (C, D, G, H; green) and BRACHYURY (E, F, G, H; red). Coexpression of the proteins will appear yellow (G, H; merge). At this stage, wild-type *Ago2* expression is restricted to the epithelial cell layer, and it does not overlap with BRACHYURY in the primitive streak. The scale bar represents 50 μm. The arrows denote the relative location of the primitive streak. The brackets indicate the approximate region of the mesoderm layer and/or the epithelial cell layer. m = mesoderm layer; ec = epithelial cell layer.

Vertebrate gastrulation initiates at e6.5 and establishes the three germ layers of the developing embryo (reviewed in [14]). During gastrulation, embryonic ectoderm (epiblast) cells are recruited to a transient embryonic structure known as the primitive streak, located on the posterior side of the embryo. At the primitive streak, the epiblast cells undergo an epithelial-to-mesenchymal transition (EMT), before migrating away from the streak and being specified as either the mesoderm or the definitive endoderm germ layers [15,16]. By e7.5, a complete mesoderm layer is formed. *Brachyury (T),* a T-box transcription factor, is expressed in the primitive streak and in the epiblast cells near the primitive streak [17,18]. To determine whether a proper primitive streak is formed in the *Ago2* mutants, we examined the expression of *T* in *Ago2* null embryos by whole-mount in situ hybridization. We found that homozygous disruption of *Ago2* results in expanded expression of *T* compared to its expression in wild-type e7.5 embryos, indicating an abnormal primitive streak in *Ago2* mutants (Figure 2A and 2B and insets). Notably, the *Ago2* mutants exhibit a variability in the expansion of *T* expression (Figure S1B and S1C), which may account for the ability of some *Ago2* mutants to escape gastrulation arrest and develop until midgestation [11]. Also consistent with previous studies is the reduced extraembryonic region in the e7.5 *Ago2* mutant embryos; this finding further suggests embryos that survive to later stages have generalized nutritional deficiencies caused by yolk sac and placental defects [11].

Previous experiments have shown that ectopic expression of *T* is sufficient to induce mesoderm formation [19], leading us to hypothesize that *Ago2* plays a role in mesoderm development. To explore this possibility, we assessed the expression pattern of another known mesoderm marker, *Tbx6* [20], and found that homozygous disruption of *Ago2* also results in an expansion of *Tbx6* expression compared with its expression in wild-type e7.5 embryos (Figure S2A and S2B). These findings, paired with the expanded *T* expression, argue for an *Ago2* function in mesoderm development.

To determine the spatial localization of *Ago2* during gastrulation, we examined its wild-type expression pattern in sectioned heterozygous *Ago2* e7.5 embryos by using antibodies against β-galactosidase (from the gene trap's *lacZ* insertion driven by the endogenous *Ago2* promoter) and BRACHYURY. We found that wild-type *Ago2* expression is restricted to the apical side of the epithelial cell layer and does not overlap with *T* in the mesenchymal cells of the primitive streak (Figure 2C, 2E, and 2G). Coupled with the fact that homozygous loss of *Ago2* results in expanded *T* expression into the epithelial cell layer (Figure 2F and 2H), these data suggest that *Ago2* could play a role in defining the primitive streak. The attenuation of *Ago2* expression as cells enter the primitive streak also raises the possibility that AGO2 plays a role in EMT. Indeed, failure to undergo proper EMT is a phenotype observed in embryos with defects in mesoderm development [21]. By contrast, because *T* is expressed throughout the epiblast of the *Ago2* mutants (Figure 2B [inset], 2F, and 2H), these mutants likely exhibit aberrant EMT because an excess of epithelial cells are being fated to become mesoderm, which ultimately could result in expanded mesoderm at the expense of the epithelial cell layer.

**Figure 3:**
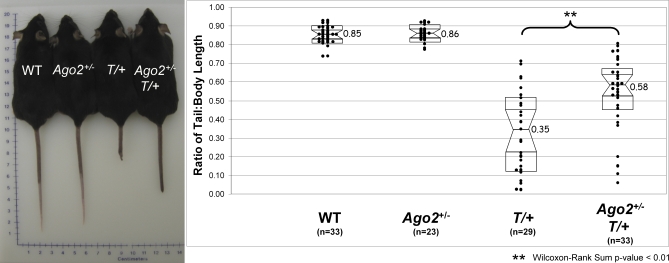
The Distribution of Tail Length for Each Genotype (A) Shown are four mice from the same litter. While the tail lengths are indistinguishable between wild-type (WT) and *Ago2* heterozygote (*Ago2^+/^* ^–^) mice, the *T* heterozygote (*T*/+) tail is reduced to approximately 30% of wild-type. In contrast, double heterozygous (*Ago2^+/^* ^–^ *T*/+) mice have tail lengths that are approximately 60% of wild-type. (B) Shown are the raw data (vertical scatterplot) overlaid with a notched-box plot. The center of the notched-box plot is the median, and the endpoints of the notches are located at the median confidence intervals. The extreme endpoints of the notched-box plot represent the 25% (lower) and the 75% (upper) quartiles of the scatter plot data. The *x*-axis shows each genotype name, and *n* is the number of mice. The *y*-axis shows the ratio of tail-to-body length. The asterisks denote that the double heterozygotes had significantly greater tail-to-body length ratios relative to single *T* heterozygotes (*p* = 0.007).

Among the mesoderm cell types induced by *T* expression are the axial and paraxial mesoderms, both of which derive the skeletal tissues that contribute to tail development in vertebrates (reviewed in [22]). In fact, the level of *T* expression correlates directly with tail length, as evidenced by the short-tail phenotype long recognized in heterozygous *T* (*T*/+) mice [12]. Remarkably, previous mapping of *T* modifier loci defined a small interval on chromosome 15 that includes the *Ago2* locus [13]. In order to genetically test whether *Ago2* could be the gene responsible for modifying the tail length in *T/+* mice, we crossed mice heterozygous for the *T* deletion with mice heterozygous for the *Ago2* disruption (*Ago2^+/^* ^–^). We plotted the ratio of tail length to body length for a quantitative comparison of heterozygous mice with double heterozygotes (Figure 3A). While the average tail-to-body ratio in both wild-type and *Ago2^+/^* ^–^ mice is approximately 0.85, the average ratio in *T/+* mice is 0.35 (Figure 3B). By contrast, the average tail-to-body ratio in double heterozygote mice is 0.58; the double heterozygotes have significantly longer tails than the *T/+* mice (*p* < 0.01). Thus, haploinsufficiency of *Ago2* results in a partial rescue of the short-tail *T/+* phenotype, demonstrating that *Ago2* is a genetic modifier of *T* expression. As an initial investigation to determine whether *Ago2* is one of the previously mapped modifiers of *T* expression [13], we searched the entire *Ago2* genomic locus (approximately 80 kb) for single nucleotide polymorphisms (SNPs) [23] and analyzed *Ago2* expression between the previously reported background strains. Remarkably, we found only one intronic SNP and that the *Ago2* expression levels are indistinguishable between the strains (unpublished data). While this might be interpreted to rule out *Ago2* as one of the previously mapped modifiers, this is a gross analysis of *Ago2* expression in whole embryos and at only a single stage of development. Indeed, our genetic data clearly show that *Ago2* is a modifier of *T* expression.

**Figure 4:**
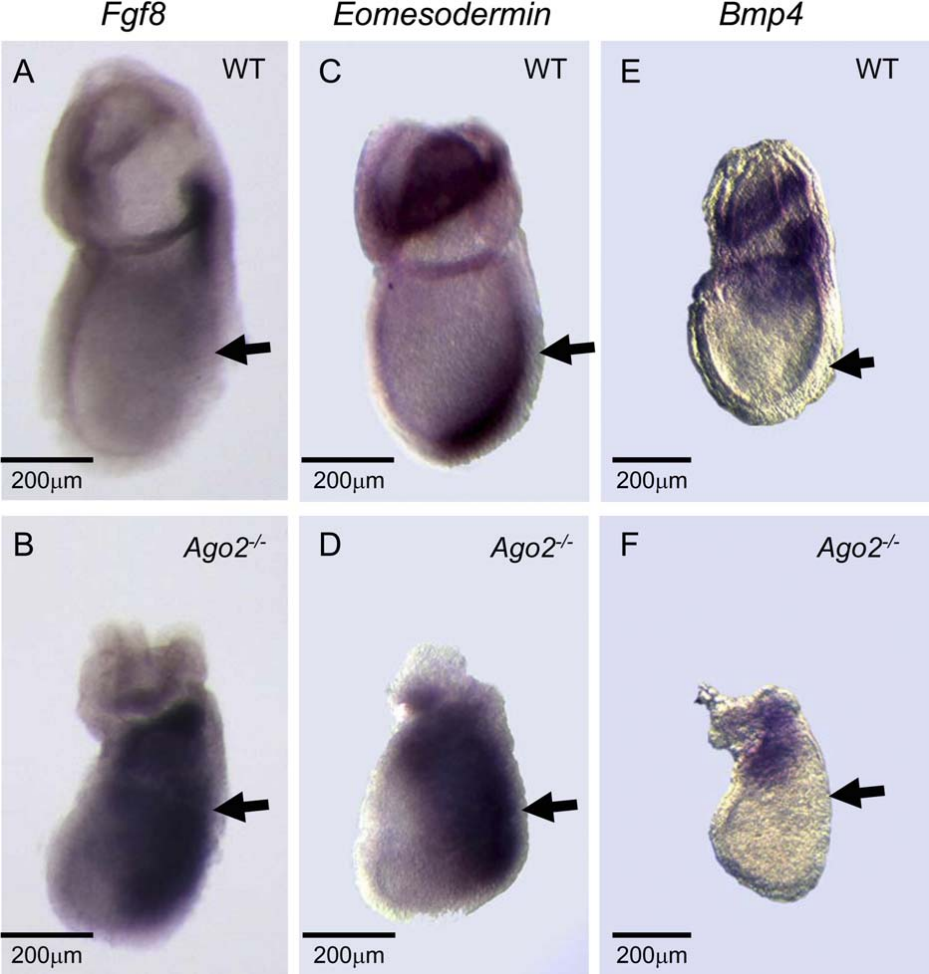
The Homozygous Disruption of *Ago2* Results in a Disruption of FGF Signaling (A−F) Whole-mount in situ hybridization using an antisense probe against *Fgf8, Eomesodermin,* or *Bmp4* on e7.5 wild-type (A, C, E) and *Ago2* ^–/–^ (B, D, F) embryo littermates. The *Ago2* ^–/–^ embryos exhibit a lateral expansion of *Fgf8* and *Eomesodermin* expression away from the primitive streak (B, D; block-arrow). In contrast, the localization of *Bmp4* expression is indistinguishable between wild-type (E) and *Ago2* ^–/–^ (F) embryo littermates. (A−D) Embryos imaged with reflective light. (E, F) Embryos imaged with reflective and transmitted light. The scale bar represents 200 μm.

These studies reveal a genetic interaction between *Ago2* and *T* and demonstrate that AGO2 mediates mesoderm development. The loss of AGO2 is known to disrupt RISC activity [11], suggesting AGO2 influences *T* expression via the micro-RNA pathway. Because the homozygous loss of *Ago2* results in expanded *T* expression into the epithelial cell layer (Figure 2F and 2H), AGO2 may utilize its “slicer” activity within the micro-RNA pathway [11] to cleave and degrade *T* transcripts expressed in the epithelial cell layer. However, in *Dicer* ^–/–^ mutants, RISC activity is disrupted upstream of *Ago2,* and these mice do not express *T* at all [24], indicating that either AGO2 is more restricted than DICER for RISC activity or the other *Argonaute* protein family members might retain a low level of functional redundancy to partially compensate for the loss of AGO2. Alternatively, AGO2 might regulate upstream inducers of *T*, such as *Bmp4, Eomesodermin, Fgfr1,* or *Wnt3a* [25–28]. Studies conducted in Xenopus laevis have demonstrated that both transforming growth factor α and FGF signaling are required to initiate *T* expression as gastrulation commences [18,29,30]. In mice, mutational analysis of the known FGF genes established that only *Fgf4* and *Fgf8* are required during gastrulation [31,32]. *Fgf4* and *Fgf8* are coexpressed throughout the primitive streak in an opposing gradient, with *Fgf8* expression highest at the posterior end of the streak and barely detectable at the anterior end. Subsequent genetic studies determined that FGF receptor 1 *(Fgfr1)* is required for the initiation of *T* expression in the posterior end of the primitive streak, suggesting that *Fgf8* is the likely ligand in this region [33]. We examined the expression of *Fgf8* in *Ago2* null embryos by whole-mount in situ hybridization and found that homozygous disruption of *Ago2* results in expanded expression of *Fgf8* compared to its expression in wild-type e7.5 embryos (Figure 4A and 4B), reminiscent of the expanded *T* expression pattern (Figure 2A and 2B). These data suggest abnormal FGF signaling causes the expanded *T* expression in *Ago2* ^–/–^ embryos.

In the mouse, direct upstream inducers of *Fgf8* are not precisely characterized, but the homozygous loss of either *Bmp4* or *Eomesodermin (Eomes)* results in failure to express both *Fgf8* and *T* [27,28]. We therefore examined the expression of *Bmp4* and *Eomes* in *Ago2*-null embryos by whole-mount in situ hybridization and found that homozygous disruption of *Ago2* results in expanded expression of *Eomes* compared to its expression in wild-type e7.5 embryos (Figure 4C and 4D), which is consistent with previous data suggesting that *Eomes* and *Fgf8* function similarly during gastrulation [28,34]. By contrast, despite the morphological differences, the localization of *Bmp4* expression is indistinguishable between *Ago2* mutants and their wild-type littermates, in that *Bmp4* expression in *Ago2* mutants remains restricted to the extraembryonic ectoderm and the proximal embryonic tissue (Figure 4E and 4F). Taken together, these data suggest that *Eomes* is an upstream inducer of *Fgf8* and that *Bmp4* is either upstream of *Eomes* or in a parallel pathway to induce *Fgf8* and *T* gene expression. Finally, as with *T,* the expansion of both *Fgf8* and *Eomes* expression in the *Ago2* mutants is varied, which again suggests a plausible explanation for those *Ago2* mutants that escape gastrulation arrest and develop until midgestation ([11]; unpublished data).

**Figure 5:**
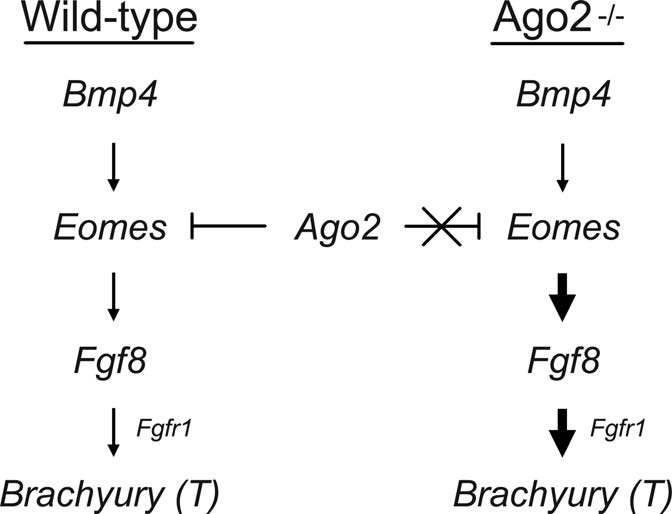
A Working Model for *Brachyury (T)* Induction at the Commencement of Mouse Gastrulation In wild-type mice, *Ago2* regulates the proper level of *Eomesodermin (Eomes)* gene expression, which ultimately induces the downstream expression of *Fgf8* and *T*. In the absence of *Ago2*, *Eomes* is not properly regulated and becomes overexpressed, resulting in the downstream overinduction of *Fgf8* and *T*. Other possible models are described in the text.

The induction of *T* expression has been studied extensively in the 15 years since the gene was cloned. These studies attribute the restricted initiation of *T* expression to morphogenic movements and cell signaling cascades by showing that disruption of these processes ultimately results in aberrant *T* expression and mesoderm development [27,28]. Coupled with earlier work in *X. laevis* demonstrating that *Bmp4* induces *Eomes* transcription [35], our data suggest a *T* induction working model in which *Bmp4* is also an upstream inducer of *Eomes* in mouse (Figure 5). At the commencement of gastrulation in wild-type embryos, *Ago2* may regulate the proper level of *Eomes* gene expression, which ultimately induces the downstream expression of *Fgf8* and *T*. In the absence of *Ago2*, *Eomes* may not be regulated properly, leading to its overexpression and a resultant downstream overinduction of *Fgf8* and *T*. Alternatively, *Ago2* may regulate an as-yet-unknown upstream inducer of *Eomes,* or *Ago2* may simultaneously have a direct influence on *Fgf8* and *T* gene expression. Because AGO2 is best known to associate with micro-RNA, it might be notable that we find computational algorithms have predicted micro-RNA binding sites in *Eomesodermin, Fgf8,* and *T* (http://microrna.sanger.ac.uk/targets/v3/), suggesting the modifying influence of *Ago2* is mediated by the micro-RNA pathway, although experimental validation of these micro-RNA binding sites awaits further study. In this case, AGO2 may utilize its “slicer” activity within the micro-RNA pathway [11] to cleave and degrade *Eomesodermin, Fgf8,* and/or *T* transcripts expressed outside the primitive streak. Distinguishing among these models will require further analysis of *Ago2*-null mice that are also null for potential upstream inducers of *T*. These possibilities notwithstanding, our findings demonstrate that AGO2 is a key factor both in the regulation of *T* expression and in mesoderm formation, placing a known component of the RNAi machinery in mammalian germ layer development.

## Materials and Methods

### Genotype and phenotype analysis.

Genomic DNA from tail or ear tissue was isolated according to standard procedures. Embryonic and full-term litters were genotyped for the *Ago2* disruption via a standard PCR procedure and the following primers: (a) 5′-CAGTGCGTCCAGATGAAGAACG-3′; (b) 5′-CCCAGGAAGATGACAGGTTG-3′; and (c) 5′-GTTTTCCCAGTCACGACGTTG-3′.

The heterozygous *T* mice **(**B10;TFLe-*a*/*a T tf*/+ *tf*/J) were purchased from The Jackson Laboratory. The *Ago2^+/^* ^–^ mice are on a congenic C57Bl/6 background, as all the mice used have been backcrossed at least ten generations onto a C57Bl/6 background. While it has been demonstrated that the background strain can affect the heterozygous *T* tail phenotype, this phenotype is not affected in strains on C57 backgrounds (e.g., C57Bl/6 and C57Bl/10; [13]). Heterozygote crosses (*T^+/^* ^–^ × *Ago2^+/^* ^–^) were set up, and the offspring (on a mix of C57Bl/6 and C57Bl/10 backgrounds) were aged 6 to 8 wk to allow for the completion of tail development. At this time, ear tissue was taken, to provide a DNA source, and each animal was subjected to a measurement of tail length as a fraction of body length. Offspring were genotyped for the *T* deletion using SYBR Green in a standard quantitative PCR procedure and the following primers: (d) 5′-CCGGTGCTGAAGGTAAATGT-3′ and (e) 5′-CCTCCATTGAGCTTGTTGGT-3′.

The resultant PCR products were quantified using the iQ5 software package and normalized against a known biallelic locus.

### Western blot analysis.

Embryos were first dissected free from the yolk sac, which was reserved for DNA extraction, then individually boiled in 30 μl of 2× Laemmli buffer before undergoing SDS 10%−PAGE. After transfer to nitrocellulose membrane, the membranes were blocked with 1% milk in PBS−0.1% Tween 20 (Blotto) and incubated with antibodies against AGO2 (Abnova) and EIF4E (BD Biosciences) for 1 h at room temperature in Blotto. Membranes were washed in Blotto and incubated with horseradish peroxidase−conjugated anti-mouse antibodies (Sigma) for 1 h at room temperature in Blotto. Membranes were washed three times in Blotto and visualized by chemiluminescence in accordance with the manufacturer's (New England Nuclear) protocol.

### In situ hybridization.

Immediately following dissection, embryos were fixed overnight in 4% paraformaldehyde (Electron Microscopy Sciences) at 4 °C. Fixed embryos were washed three times in PBS, dehydrated through a methanol series (25%, 50%, 75%, 2× 100%), and stored at −20 °C. In situ hybridizations were performed on whole-mount embryos, as described [36,37]. Antisense riboprobes were synthesized from *Brachyury, Fgf8, Eomesodermin,* and *Bmp4* cDNA-containing plasmids using a digoxigenin-UTP labeling kit (Roche). Digoxigenin-labeled compounds were detected using alkaline phosphatase−conjugated antidigoxigenin (Roche). Whole-embryo images were captured using a dissection scope (Zeiss Stemi) with attached camera (Zeiss AxioCam MRc). Following in situ hybridization, embryos were paraffin embedded using a standard protocol. Then 10-μm sections were dried to positively charged slides (Surgipath). Dried sections were deparaffinized and hydrated by standard procedures. Sections were imaged using a Zeiss Axioskop with attached camera (SPOT, Diagnostic Instruments, Inc.).

### Immunohistochemistry.

Immediately following dissection, embryos were fixed for 2 to 3 h in a 6:3:1 ratio of 100% EtOH/37% formaldehyde (Fisher)/100% acetic acid (Fisher) at 4 °C. Fixed embryos were washed 3× in PBS and were paraffin embedded using a standard protocol. Then 10-μm sections were dried to positively charged slides (Surgipath). Dried sections were deparaffinized and hydrated by standard procedures, before blocking endogenous peroxidases in 100% methanol/3% hydrogen peroxide for 10 min at room temperature. Sections were rinsed with water and PBS prior to antigen retrieval using a standard procedure (Dako). Following PBS washes, sections were blocked in 5% donkey serum/2% BSA for 1 h at room temperature. Blocked sections were incubated overnight with primary antibodies against *T* (Santa Cruz) and β-galactosidase (Cappel) at 4 °C. Sections were then rinsed in PBS and incubated for 1 h with the corresponding secondary antibodies (Invitrogen) at room temperature. Sections were rinsed in PBS and coverslips were mounted with *n*-propyl gallate (Sigma). Confocal imaging was performed using the ×20 objective lens and a Zeiss LSM 510 confocal microscope system (Figure 2).

### Statistical analysis.

We initially applied a Shapiro-Wilks test to our data to determine whether tail-to-body length ratios followed a normal distribution. When results indicated that the distribution was not normally distributed (*p* = 0.0043), we applied a nonparametric test of independent samples (Wilcoxon rank-sum) to assess differences in the tail-to-body length ratios between single (*T/+; n* = 29) and double (*T/+ Ago2* ^+/–^; *n* = 33) heterozygotes. The double heterozygotes were found to have significantly greater tail-to-body length ratios compared with single heterozygotes (*p* = 0.007). The endpoints of notches on the notched-box plot are located at the median ± 1.58 (IQR/square root of *n*), where IQR represents the interquartile range and *n* is the subgroup sample size [38].

## Supporting Information

Figure S1 The Homozygous Disruption of *Ago2* Results in a Variable Expansion of *T* Expression (A−C) Whole-mount in situ hybridization using an antisense probe against *T* on e7.5 wild-type (A) and *Ago2* ^–/–^ (B, C) embryos. The *Ago2* ^–/–^ embryos exhibit an expansion of the primitive streak (block-arrow). The expansion can be classified as either partial [(B); 9/17 *Ago2* ^–/–^ mutants] or profound [(C); 8/17 *Ago2* ^–/–^ mutants]. The scale bar represents 200 μm.
(1.4 MB PDF)

Figure S2 The Homozygous Disruption of *Ago2* Results in an Expansion of *Tbx6* Expression (A, B) Whole-mount in situ hybridization using an antisense probe against *Tbx6* on e7.5 wild-type (A) and *Ago2* ^–/–^ (B) embryos. The *Ago2* ^–/–^ embryos exhibit an expansion throughout the embryo. The scale bar represents 150 μm.
(57 KB PDF)

## Abbreviations

EMT: epithelial-to-mesenchymal transition
FGF: fibroblast growth factor
RISC: RNA-induced silencing complex
RNAi: RNA interference
SNP: single nucleotide polymorphism
