# Supplementary material for: Argonaute2 Is Essential for Mammalian Gastrulation and Proper Mesoderm Formation
Source: PLoS Genet. 2007 Dec 28;3(12):e227. doi: 10.1371/journal.pgen.0030227 (PMC2323323; doi:10.1371/journal.pgen.0030227)
Supplement: Figure S1 — (A−C) Whole-mount in situ hybridization using an antisense probe against T on e7.5 wild-type (A) and Ago2 –/– (B, C) embryos. The Ago2 –/– embryos exhibit an expansion of the primitive streak (block-arrow). The expansion can be classified as either partial [(B); 9/17 Ago2 –/– mutants] or profound [(C); 8/17 Ago2 –/– mutants]. The scale bar represents 200 μm. (1.4 MB PDF) [file pgen.0030227.sg001.pdf]

Supporting Figure 1
The homozygous disruption of Ago2 results in a variable expansion of T expression. (A-C)
Whole-mount in situ hybridization using an antisense probe against T on e7.5 wild-type (A) and Ago2-/- (B,C) embryos. The Ago2-/- embryos exhibit an expansion of the primitive streak (block-arrow). The expansion can be classified as either partial (B; 9/17 Ago2-/- mutants) or profound (C; 8/17 Ago2-/- mutants). The scale bar represents 200 µm.

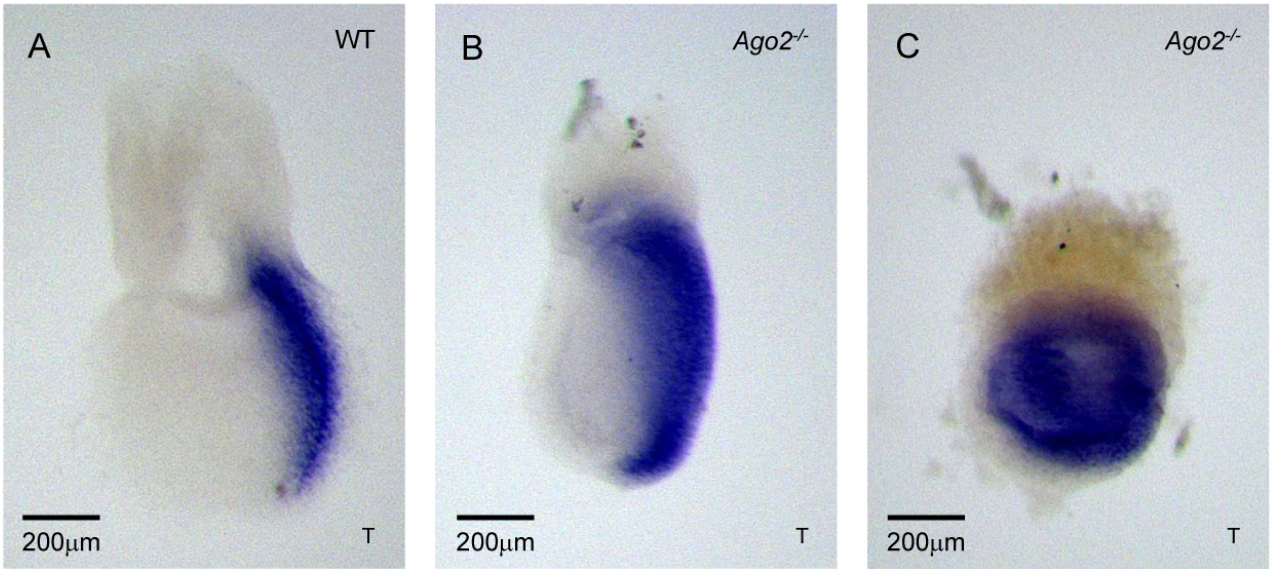

Partial expansion

Profound expansion
