# Supplementary material for: Argonaute2 Is Essential for Mammalian Gastrulation and Proper Mesoderm Formation
Source: PLoS Genet. 2007 Dec 28;3(12):e227. doi: 10.1371/journal.pgen.0030227 (PMC2323323; doi:10.1371/journal.pgen.0030227)

Supporting Figure 2

The homozygous disruption of *Ago2* results in an expansion of *Tbx6* expression. (A-B) Whole-mount *in situ* hybridization using an antisense probe against *Tbx6* on e7.5 wild-type (A) and *Ago2-/-* (B) embryos. The *Ago2*-/- embryos exhibit an expansion throughout the embryo. The scale bar represents 150 μm.

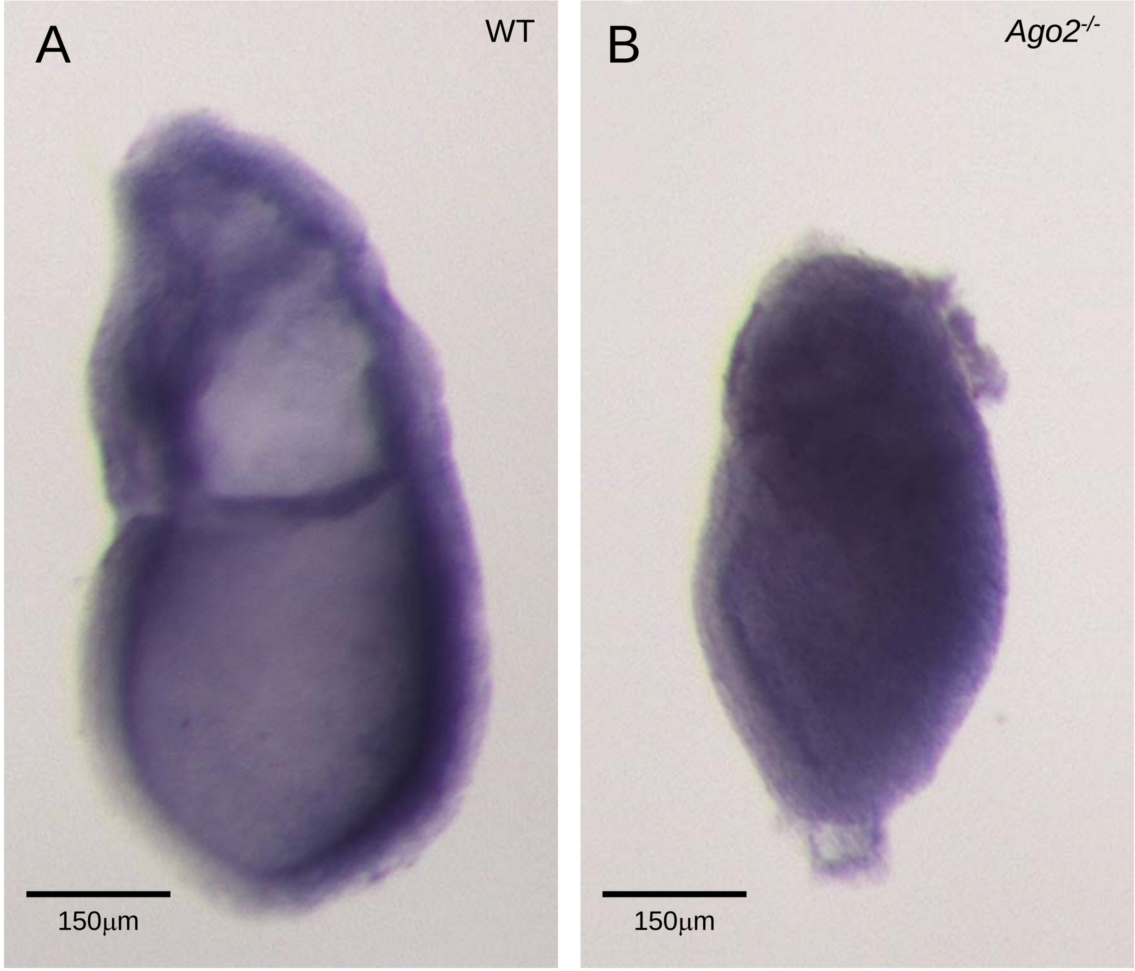

Supplement: Figure S2 — (A, B) Whole-mount in situ hybridization using an antisense probe against Tbx6 on e7.5 wild-type (A) and Ago2 –/– (B) embryos. The Ago2 –/– embryos exhibit an expansion throughout the embryo. The scale bar represents 150 μm. (57 KB PDF) [file pgen.0030227.sg002.pdf]
